# Integrating Medication Therapy Management (MTM) Services Provided by Community Pharmacists into a Community-Based Accountable Care Organization (ACO)

**DOI:** 10.3390/pharmacy5040056

**Published:** 2017-10-16

**Authors:** Brian Isetts

**Affiliations:** Department of Pharmaceutical Care & Health Systems, University of Minnesota College of Pharmacy, 308 Harvard St., SE Minneapolis, MN 55455, USA; isett001@umn.edu; Tel.: +1-612-624-2140

**Keywords:** community pharmacist interventions, value-based care models, medication therapy management

## Abstract

(1) Background: As the U.S. healthcare system evolves from fee-for-service financing to global population-based payments designed to be accountable for both quality and total cost of care, the effective and safe use of medications is gaining increased importance. The purpose of this project was to determine the feasibility of integrating medication therapy management (MTM) services provided by community pharmacists into the clinical care teams and the health information technology (HIT) infrastructure for Minnesota Medicaid recipients of a 12-county community-based accountable care organization (ACO). (2) Methods: The continuous quality improvement evaluation methodology employed in this project was the context + mechanism = outcome (CMO) model to account for the fact that programs only work insofar as they introduce promising ideas, solutions and opportunities in the appropriate social and cultural contexts. Collaborations between a 12-county ACO and 15 community pharmacies in Southwest Minnesota served as the social context for this feasibility study of MTM referrals to community pharmacists. (3) Results: All 15 community pharmacy sites were integrated into the HIT infrastructure through Direct Secure Messaging, and there were 32 recipients who received MTM services subsequent to referrals from the ACO at 5 of the 15 community pharmacies over a 1-year implementation phase. (4) Conclusion: At the conclusion of this project, an effective electronic communication and MTM referral system was activated, and consideration was given to community pharmacists providing MTM in future ACO shared savings agreements.

## 1. Introduction

The aim of this project was to integrate medication therapy management (MTM) services provided by community pharmacists into the clinical care teams and the health information technology (HIT) infrastructure for Medicaid recipients of a 12-county community-based accountable care organization (ACO). Southern Prairie Community Care (SPCC) of Southwest Minnesota invited colleagues from the University of Minnesota College of Pharmacy to collaborate on integrating medication management services into the redesigned care delivery and financing system of the 12-county SPCC Accountable Care Organization. This was a novel opportunity to engage community pharmacists in a feasibility test of change related to value-based care model integration and financing. Funding for this feasibility study was obtained from the Community Pharmacy Foundation.

The U.S. healthcare system is evolving from fee-for-service reimbursement to an outcomes-based payment system based on reductions in total cost of care and improvements in quality of care performance measures of the Centers for Medicare & Medicaid Services (CMS) Quality Payment Program (QPP) [1].

In this feasibility study, there was an existing reimbursement system in place for the provision of MTM services by pharmacists through the Minnesota Medicaid Program. This existing reimbursement mechanism helped to address a common challenge of providing MTM services. Other common challenges to the provision of MTM services include collaborative relationships with prescribers, billing difficulties, inadequate management support for MTM, and technology barriers such as access to patients’ medical records and bi-directional exchange of health information [2].

The SPCC community-based care system has engaged multiple providers, payers and stakeholders since 2006 in building capacity to increase local control around service decisions made for vulnerable people living in the 12-county region. Local pharmacists contributing to SPCC work groups and planning committees during the stakeholder engagement process provided outcomes research data on the impact of MTMS provided by pharmacists, convincing SPCC decision-makers of the need to help patients confidently manage their medications. Pharmacist engagement in the SPCC stakeholder planning process focused attention on the need for building systems to improve the effective and safe use of medications and to reduce drug-related morbidity and mortality.

SPCC is accountable for the medical, facilities, and pharmacy costs of over 20,000 attributable Minnesota Medicaid recipients living in the 12-county region, and there are approximately 36,000 Medicaid recipients in the 12-county SPCC area. Attribution is a method for assigning patients to an ACO based on the utilization history of a patient. Medicaid recipients who are not attributable to the SPCC network typically receive care outside the 12-county area or outside of the SPCC network. It is noted that pharmacy costs constituted nearly one-fourth of total expenditures for SPCC Medicaid recipients, which is well above national averages for pharmaceutical expenditures reported by the Commonwealth Fund based on Organization for Economic Cooperation and Development (OECD) Health Data [3].

The objectives of this project were to: (1) ensure service level expectations of MTM services provided by community pharmacists; (2) equip pharmacists to function effectively in the SPCC healthcare teams; and (3) integrate the electronic documentation of community pharmacists providing MTMS into the SPCC HIT infrastructure. The significance of this initiative relates to community pharmacist integration in new care delivery models supporting the Medicare Access and Chip (Children’s Health Insurance Program) Reauthorization Act (MACRA) reimbursement programs.

## 2. Materials and Methods

The methodological approach used in this project combined evidence-based medicine with the science of continuous quality improvement. The relationship of evidence-based medicine to the science of quality improvement can be described as consistently doing the right thing right. Pawson and Tilley pointeyearbookd out that evidence-based medicine is deeply vested in experimental design using an OXO evaluation approach of: observe a system (O), introduce a perturbation/intervention (X) to some participants but not others, and then observe again (O) [4]. Dr. Don Berwick, former CMS Administrator and champion of the Science of Quality Improvement, has noted that the OXO paradigm most commonly applied in the traditional toolkit of evidence-based medicine is, “a powerful, perhaps unequaled, research design to explore the efficacy of conceptually neat components of clinical practice—tests, drugs, and procedures. For other crucially important learning purposes, however, it serves less well” [5].

Previous research projects have replicated favorable clinical and economic outcomes of pharmacist integration in comprehensive, team-based medication management [6,7,8,9,10,11]. The evaluation question is no longer *if* pharmacist integration in care model innovation works, but *how* do we make it work more effectively and efficiently? The introduction of interprofessional and interdisciplinary systems for establishing a rational medication use system in which patients routinely achieve their goals of therapy with zero tolerance for preventable medication harms is a complex, multicomponent intervention—essentially a process of social change.

An alternative evaluation approach applied in this project was the context + mechanism = outcome (CMO) model. This evaluation approach accounts for the fact that programs only work insofar as they introduce promising ideas, solutions and opportunities in the appropriate social and cultural contexts [4,5]. One example of the CMO model in use on a large national scale involved concerted rapid cycle quality improvement to reduce medical harms and to decrease readmissions. Results from collaborations among federal partners and external stakeholders indicate that there were 2.1 million fewer patient harms and 87,000 deaths prevented, saving $19.8 billion in costs in the CMS Innovation Center—Partnership for Patients initiative. These improvements included an 8% reduction in Medicare 30-day fee-for-service readmissions and a 15% decrease in hospital acquired adverse drug events [12,13].

### 2.1. Study Design

This was a 24-month feasibility study that included an 8-month community outreach phase, a 12-month implementation phase and a 4-month evaluation phase, spanning from October 2014–September 2016. The study design focused on three major objectives. The first objective relates to understanding the care capacity of pharmacists working in the 12-county region, and coordinating service level expectations of MTMS provided by pharmacists. The second objective was preparing pharmacists to function effectively in high performing teams. The third objective was integrating the electronic documentation of community pharmacists providing MTMS into the SPCC HIT infrastructure.

#### 2.1.1. Objective 1

Ensuring service level expectations of pharmacists providing MTMS was the responsibility of the Principal Investigator working in collaboration with colleagues from the MedEdge Rx (formerly UPlan) MTM Network. The MedEdge Rx Network coordinated communications among credentialed providers. Prior to project inception, it is noted that pharmacists throughout the 12-county region were formally introduced to the SPCC Accountable Care Organization initiative in December 2013 at a local Minnesota Pharmacists Association Pharmacy Night in which the SPCC CEO served as the keynote presenter.

This project represented an opportunity to build a rational medication use system within redesigned care delivery intended to integrate the resources of pharmacists living and working in the SPCC communities. The pharmacy workforce profile of the 12-county SPCC area includes approximately 80 pharmacists working in 34 community pharmacies, 15 critical access hospitals, three regional hospitals, and 25 area clinics. The 12 Minnesota counties in the SPCC area include (from North to South), Swift, Kandiyohi, Chippewa, Yellow Medicine, Lincoln, Lyon, Redwood Falls, Murray, Cottonwood, Rock, Nobles, and Jackson County. Pharmacists working in 15 community pharmacies and in 2 clinic pharmacies were credentialed providers of the MedEdge Rx MTM Network providing MTMS consistent with State of Minnesota MTM requirements at project inception [14]. All other pharmacists in the SPCC area were contacted in-person and/or by telephone encouraging them to complete MTMS training to become recognized providers by the State of Minnesota Department of Human Services (Minnesota DHS) and enrolled in the MedEdge Rx MTM Network.

Pharmacists in the 12-county region were provided with the opportunity to take Minnesota DHS-approved MTM training programs, if they had not already completed training. The MedEdge Rx MTM Network coordinated oversight for credentialing pharmacists in the SPCC region, providing access to peer mentors, and assessing and monitoring quality of care delivered by pharmacists. An Exempt Category 4 Institutional Review Board (IRB) application was reviewed by the University of Minnesota Human Research Protection Program [15].

#### 2.1.2. Objective 2

This project was designed to equip MTM pharmacists to function effectively in high performing teams for making valuable contributions toward helping patients achieve their drug therapy treatment goals and resolve drug therapy problems. Pharmacists, healthcare teams, and patient advisors across the SPCC area were brought together to build relationships and capacity utilizing portions of the primary care version of Team Strategies and Tools to Enhance Performance and Patient Safety (TeamSTEPPS) [16]. Modified TeamSTEPPS training programs were offered on multiple times/dates so that pharmacists and SPCC providers could have flexibility in attending training programs. A TeamSTEPPS Master Trainer was utilized to lead this program with support from the Principal Investigator.

In addition, a series of periodic half-day workshops were produced to bring community care team members together for the purpose of accelerating progress toward community-based medication management collaborations. Each workshop program included an overview of comprehensive medication management generally and the SPCC MTM Network specifically, as well as small workgroup sessions focusing on areas of care coordination. Topics of focus throughout the SPCC MTM Workshop series included, conducting co-visits, promoting the MTM service to SPCC recipients, and clinical topics such as building community-based systems to assist patients with cardiovascular and mental health needs.

#### 2.1.3. Objective 3

Pharmacist integration into health information technology (HIT) systems and infrastructure is vitally important to effective and efficient team-based care, although access to critical medical information has been historically challenging to obtain for community pharmacists. The third objective of this project was integrating the electronic documentation of community pharmacists providing MTMS into the SPCC HIT infrastructure. Important steps in addressing this objective included:

- Creating the legal and regulatory scaffolding supporting community pharmacist integration in the SPCC HIT system,
- Providing community pharmacists with access to the recently-released national encryption standard for securely exchanging clinical healthcare data and Protected Health Information (PHI) known as Direct Secure Messaging, and,
- Developing processes to help pharmacists more effectively identify SPCC attributable Medicaid recipients for MTM services.

The legal and regulatory infrastructure for pharmacist participation in the SPCC HIT system relates to meeting standard health provider conditions of participation including HIPAA-compliant Provider Participation and Business Associate Agreements. Access to Direct Secure Messaging ensures community pharmacist compliance with the electronic health record technical specifications of the Medicare Access and CHIP Reauthorization Act (MACRA). In addition, pharmacist access to accurate lists of recipients that are both eligible for reimbursement through the Minnesota Medicaid MTM Program, and attributable to the SPCC network would be beneficial in prioritizing SPCC recipients for MTM services. 

Southern Prairie Community Care is officially recognized by the State of Minnesota as a Health Information Organization (HIO) under Minnesota Statutes § 2015 62J.4981 [17]. At the time of project inception, SPCC employed the use of a company by the name of Sandlot Solutions, Inc., to serve as its HIT integration vendor, consistent with State of Minnesota technical HIT specifications. The University of Minnesota executed a subcontractor agreement with Santa Rosa Consulting, Inc., the parent company of Sandlot Solutions Inc., to conduct a technology survey of SPCC MTM network pharmacies and to guide the pharmacy HIT integration process.

Integrating pharmacists into the SPCC HIT infrastructure began with a technology survey to assess interoperability and compliance with meaningful use standards. Results of the technology survey were then used to structure technology site visits to each of the SPCC MTM network pharmacies. Once interoperability capabilities were established, original project plans called for the health integration vendor to assist in delivering a Webex training session on procedures and protocols for exchanging electronic health information. The intent of this project was to provide pharmacies with read-only access to medical records of SPCC attributable Medicaid recipients, and if feasible, select one pharmacy to test the bi-direction transfer of medical records and MTM documentation.

## 3. Results

Final project results were significantly influenced by major SPCC events and challenges. These challenges were related to the SPCC HIT master integration plan and recurring key personnel transitions. These challenges are briefly discussed below as a pretext to presenting project results.

The most significant challenge occurred within the SPCC HIT integration process. As SPCC embarked upon an ambitious goal of creating a common medical records platform across providers, health systems and electronic medical record systems, a number of technical problems were encountered. The goal of creating a common medical record platform for physicians, clinics and MTM pharmacies remained elusive. Early during this project, it became evident that the SPCC timeline for HIT integration was fraught with challenges. The original plan called for HIT integration of physician practices and clinics, followed by SPCC MTM pharmacists and pharmacies. Then, early in 2016, the SPCC HIT Integration Vendor, Sandlot Solutions, abruptly announced it was going out of business [18]. The other significant challenge relates to recurring key personnel losses. Over the course of this project, numerous Care Coordination Integrators were employed and then moved on to other opportunities, and the SPCC Executive Director also departed. 

### 3.1. Feasibility Evaluation

As described in the Methods section, the program evaluation question applied to this feasibility study is not *if* pharmacist integration in care model innovation works, but *how* it works more effectively and efficiently. Pharmacist integration in health system settings of hospitals, clinics, and nursing homes has been in progress for many years. However, community pharmacist integration in emerging community-based care delivery systems is a relatively recent innovation. Therefore, this program evaluation assessed feasibility from both a near-term and a long-term perspective.

The near-term feasibility evaluation utilized the three study objectives of; ensuring service level expectations for MTMS, pharmacist engagement in community-based healthcare teams, and integration into the SPCC Health Information Exchange (HIE). Peer review of MTMS documentation for the 32 SPCC recipient referrals revealed that Minnesota Department of Human Services documentation standards were met for these recipients [14]. Interviews conducted with MTM pharmacists and SPCC personnel at the conclusion of the study indicated that pharmacists at seven of the 17 SPCC MTM sites collaborated with community-based healthcare providers to provide MTM services. In addition, the feasibility of integration in the SPCC HIE system was, at least partially achieved, through pharmacist access to the SPCC Direct Secure Messaging System designed for secure communications among community-based providers.

Long-term feasibility was made possible by determining the number of SPCC MTM pharmacists continuing to care for SPCC recipients one year after study conclusion. This study was conducted from October 2014–September 2016. Post-study interviews were conducted with MTM pharmacists and SPCC personnel in September 2017 indicating that five of the 15 SPCC community pharmacy MTM sites, and both of the clinic-based MTM sites, were continuing to collaborate with health team members in their communities to provide MTM services. Additional results are presented below within each of the three study objectives.

Objective 1: Ensuring Service Level Expectations of Pharmacists Providing MTMS

### 3.2. Pharmacist Credentialing and Collaboration with MN Department of Human Services (DHS)

The University of Minnesota Human Research Protection Program served as the Institutional Review Board (IRB) for this project, and an Exempt Category 4 IRB application was submitted and reviewed in September of 2014. The goal for this project at inception was to have pharmacists at 12 sites approved by MN DHS as MTM providers. There were 24 pharmacists at 17 sites recognized by the Minnesota Department of Human Services and credentialed by the MedEdge Rx Network as MTM providers. The distribution of 17 sites includes 15 community pharmacies, one clinic pharmacy owned by a community pharmacy, and one pharmacy in a family practice clinic. And the distribution of community pharmacies includes 12 sites owned by regional chain pharmacies, and 4 independent pharmacy sites.

### 3.3. MTM Billing

The Minnesota Medicaid—Resource-based Relative Value Scale reimbursement system was applied to the care delivered by pharmacists in this project. Of the 20,000 attributable SPCC recipients, the approximate distribution of recipients across health plans included, Blue Cross/Blue Shield (BCBS) managed care Medicaid (~50%), other managed care Medicaid plans (~30%), and MN Department of Human Services (DHS) fee-for-service Medicaid (~20%). MTM claims were submitted as a medical care benefit on an electronic CMS Form 1500 platform, which is the same claims format used for all other health care services. Procedures for submitting MTM claims for MN DHS fee-for-service recipients through the MN-eCONNECT system have been used for the past 10 years. It is noted that SPCC, Blue Cross/Blue Shield and University of Minnesota researchers collaborated to facilitate MTM contracting, and to provide three WebEx training programs for BCBS on-line MTM billing. Reimbursement levels and other economic and clinical data for the 32 SPCC referred recipients receiving MTM services was not an objective of this feasibility project.

Objective 2: Equipping Pharmacists to Function Effectively in SPCC Healthcare Teams

### 3.4. Interprofessional Team Training and Integration

The effective delivery of MTM provided by pharmacists is dependent on collaborations with a patient’s health care team. Interprofessional collaboration represents the “high-touch” element of high-touch/high-tech integration. A series of interprofessional training programs were conducted throughout the course of this project. As part of the community outreach phase, a general MTM Informational Webinar was provided to SPCC community care teams. Then, an interprofessional training program was held that brought pharmacists and SPCC healthcare providers together to start the process of MTM collaboration. During the implementation phase, an SPCC Care Coordinator/MTM Pharmacist workgroup meeting was held to plan the MTM referral process for SPCC recipients, followed by a Care Coordinator workshop and then an SPCC Behavioral Health MTM workgroup meeting.

### 3.5. Recipient Identification Including MTM Data Driven Intervention Strategy for High Priority Recipient Enrollment

The SPCC ACO presented their proposed Access Design Requirements to the Minnesota Department of Human Services (MN DHS) in February 2016. Pursuant to this formal review and approval, SPCC began preparing the attribution file, or list of eligible recipients, for all healthcare providers across the SPCC network. SPCC ACO leaders then developed a Data Driven Intervention Strategy (DDIS) using recipient’s prior medical, hospital and pharmacy claims in the previous year to help them prioritize recipients for MTM referrals. The claims-based data elements for DDIS referrals included recipients in the top 20% of total cost of care or total pharmacy expenditures, or recipients with three or more hospital or emergency department visits over a six-month period. 

The original plan to distribute each pharmacy’s list of attributable MTM recipients was delayed on several occasions pending DDIS access design reviews by MN DHS. SPCC prioritized MTM recipient referrals based on total health expenditures, medication expenditures and previous hospitalization and emergency room visits. A total of 32 SPCC recipients received MTM services pursuant to referrals at five community pharmacy sites.

Objective 3: Integrating Electronic Documentation in the SPCC HIT Infrastructure

### 3.6. Health Information Technology (HIT) Integration

Integrating community pharmacists into the HIT infrastructure of the 12-county Southern Prairie Community Care (SPCC) Accountable Care Organization was a key objective of this project. One of the first HIT integration tasks completed early in the project was to have each MTM pharmacy execute HIPAA-compliant Business Associate Agreements and Health Information Exchange Agreements with SPCC. The University of Minnesota then contracted with Santa Rosa Consulting (the parent company of Sandlot Solutions) to assist in the HIT integration process. Santa Rosa Consulting was the same HIT consulting firm hired by SPCC to facilitate exchange of HIT in clinics and health systems across the 12-county ACO.

At the outset of the project there was an ambitious HIE integration plan for community pharmacists that included the following steps:

1 Design of a technology survey for SPCC pharmacies providing MTM services,
2 Technology site visits to SPCC pharmacies with the Health Information Exchange (HIE) Integration Vendor,
3 WebEx training to prepare pharmacies for receiving read-only access to the SPCC HIE infrastructure via a web based portal,
4 Development of technical design specifications for bi-directional data interchange between a pilot MTM pharmacy site and a central SPCC medical exchange platform.

Santa Rosa Consulting met their Step 1 and 2 statements of work. Results of the technology survey revealed that there would need to be a substantial investment to integrate the patient care documentation systems of SPCC MTM pharmacies to facilitate bi-directional data exchange. The primary challenge to bi-directional data exchange of SPCC MTM documentation was because pharmacist care plan HIE standards were not yet established at the time of this study. It is noted that the Pharmacist eCare Plan public/private initiative was recently launched to develop a standardized, interoperable document for exchange of medication management care plans and goals of therapy for pharmacists working in multiple environments [19].

However, the Step 3 and 4 objectives were not met due to insolvency of the HIE Vendor, and funds were reallocated for two alternative HIE integration approaches. A new health care communications portal advocated by the U.S. Department of Health & Human Services, Office of the National Coordinator for Health Information Technology (ONC), known as Direct Secure Messaging, was deployed throughout the SPCC region [20]. Direct Secure Messaging facilitates communications directly between health care providers, rather than relying on facsimile transmission via telephone lines. Each MTM pharmacy’s startup costs for Direct Secure Messaging was paid by reallocating funds originally intended for the HIT Integration Vendor. A second approach was for SPCC MTM pharmacies to utilize Business Associate Agreements to gain direct access to their local health systems electronic medical record (EMR) system. At the conclusion of the project, one SPCC MTM community pharmacy was successful in securing read-only access to their local health system’s EMR system.

### 3.7. Qualitative Indicators of Care Model Integration

There were a number of new collaborations and serendipitous events that occurred during this project serving as qualitative indicators of care model integration. As community pharmacists, social service providers, mental health professionals, and community health workers began collaborating around a new community-based model of care; there was evidence of team-based medication management similar to characteristics of integrated health systems employing pharmacists on health teams. Some of these indicators included:

- Pharmacist/Integration Coordinator MTM co-visits including one with a recipient utilizing 44 Emergency Department (E.D.) visits over a 6-month period. The result was that this recipient had no further E.D. visits over the implementation and evaluation phases of this project. There were two direct quotations from SPCC stakeholders related to the care of this recipient that are noteworthy. The Integration Coordinator performing the MTM co-visit noted that, ‘I didn’t know that pharmacists had this type of training to help patients with their medication problems.” The other quote came from the Emergency Department Coordinator at a meeting six months after the MTM co-visit lamenting, ‘We haven’t seen (this recipient) in six months and we thought that (this person) had passed away.’
- MTM pharmacists at a community pharmacy site located inside a medical clinic were invited to join the clinic’s Primary Care Provider Department with dedicated office space to provide MTM services. In addition, this invitation created the opportunity to create a post-graduate pharmacy residency experience at this site. During a Principal Investigator site visit shortly after the MTM pharmacist joined this practice, a primary care provider described elation in this newfound collaboration noting that, ‘We always had pharmacists on our teams during my residency training in large health systems, and I never thought this was possible in a small rural setting.’
- Collaborations with the local Health Department to execute a Centers for Disease Control and Prevention (CDC) cardiovascular health outreach grant in which the Community Health Worker was co-located at the MTM Community Pharmacy.
- Placement of an SPCC Integration Coordinator inside one of the community pharmacy MTM sites.
- Placement of an SPCC Somali Cultural Liaison at one of the MTM community pharmacy sites located in a grocery store.
- The appointment of an SPCC MTM Pharmacist to serve on the Southern Prairie Center for Community Health Improvement Board of Directors.

## 4. Discussion and Future Implications

Although extenuating circumstances that occurred when the SPCC HIT Integration Vendor went out of business coupled with turnover of key SPCC personnel were disappointing, there are a number of highlights and lessons learned that are expected to be helpful in the future. Qualitative indicators and anecdotal reports from the SPCC MTM referrals were encouraging. As community pharmacists in the 12-county SPCC region strive to provide MTM services, discussions have progressed on including SPCC MTM pharmacies in shared savings agreements in the future. Minnesota was one of the first six CMS State Innovation Model (SIM) sites selected to accelerate progress towards value-based reimbursements. And shared savings agreements represent a bridge to global payment systems for achieving better care and better health at lower cost.

This feasibility study was designed to address multiple barriers to pharmacist integration in community-based care teams. As noted previously, common challenges to the provision of MTM services include reimbursement, collaborative relationships with prescribers, billing difficulties, inadequate management support for MTM, and technology barriers such as access to patients’ medical records and bi-directional exchange of health information [2]. This study helped community pharmacies by incorporating reasonable reimbursement rates for MTM services, building collaborative relationships with prescribers, streamlining MTM billing and contracting, and addressing technological barriers to a limited extent. 

It became apparent that inadequate management support was the common factor among the 10 community pharmacies that were unsuccessful in this project. The context + mechanism = outcome (CMO) model applied in this study suggests that programs only work insofar as they introduce promising ideas, solutions and opportunities in the appropriate social and cultural contexts [4,5]. The five successful sites provided adequate management support by changing the cultural context of their community pharmacy business model so that the business of patient care could coexist and complement the business of prescription dispensing. This management support included reorienting all community pharmacy personnel to the business of patient care, allocating pharmacist time to caring for patients, and expanding pharmacy technician responsibilities to assist with patient recruitment, scheduling, billing and documentation. This means that future efforts to assist community pharmacies in building patient care practices will need to guide the implementation of effective change management. In other words, removing all other barriers to pharmacist integration in community-based care teams is insufficient without guiding management support for effective change management.

It is important to highlight the relationship of this project to the new physician-focused reimbursement system of the Medicare Access and CHIP Reauthorization Act (MACRA). The two MACRA reimbursement tracks of the Merit-based Incentive Payment System (MIPS) and the Advanced Alternative Payment Models (APM) are founded on achieving Quality Payment Program benchmark measures in the four broad categories of Quality, Improvement Activities, Advancing Care Information, and Cost. The Physician Quality Reporting System served as a backbone of MACRA, and over one-half of the nearly 300 MIPs and APM Program measures are dependent on the effective or safe use of appropriately indicated medications. The profession of Pharmacy has established an extensive research portfolio demonstrating the impact of medication therapy management services on economic, clinical, and humanistic outcomes. And steps taken in this project to integrate community pharmacists in a community-based ACO are expected to help accelerate collaborations with physicians to achieve MACRA benchmarks.

The elusive goal of Health Information Integration (HIE) was prominent in this feasibility study. Although the ambitious objective of bi-directional HIE was not achieved, the integration of MTM pharmacists into the SPCC Direct Secure Messaging infrastructure has the potential to accelerate use of the Pharmacist eCare Plan standards, when fully developed [19]. The Pharmacy HIT Collaborative has championed a national focus on ensuring the meaningful use of standardized electronic health records (EHR) that supports safe, efficient, and effective medication use, continuity of care, and providing access to the patient-care services of pharmacists with other members of the interprofessional patient care team [21]. The experiences and results of this project can help other health systems and community pharmacies understand the steps and challenges that will need to be addressed to achieve this Pharmacy HIT Collaborative vision.

Assessing the need for community pharmacist engagement in value-based healthcare delivery and financing is summarized in a White Paper produced under contract from the Pharmacy Quality Alliance. *Applying Value-based Incentive Models within Community Pharmacy Practice* presents the landscape for engaging community pharmacists in improving health care quality through innovative care delivery and payment models. Key concepts applied in this PQA White Paper included arrangements for pharmacists/pharmacies to share savings with ACO providers, and implementing value-based insurance programs in community pharmacies [22].

### Limitations

One potential limitation of this feasibility study is that it was not designed to evaluate clinical or economic outcomes of MTM services. As noted in the Methods section, previous results of research projects demonstrating favorable clinical and economic outcomes of pharmacist integration in comprehensive, team-based medication management has shifted the evaluation question to *how* we can make this integration more effective and efficient. The fact that an existing reimbursement system was in place for the provision of MTM services by pharmacists through the Minnesota Medicaid Program facilitated the design of this feasibility study.

It is noted that the provision of MTMS for children was not a component of this feasibility study. Although pharmacists may provide MTMS for Minnesota Medicaid recipients under the age of 18, this patient population was not a focus of the SPCC attributable ACO population. Future feasibility studies related to the provision of MTMS for children in evolving MACRA care delivery and financing systems is warranted.

Although reducing the burden of ineffective medication use and drug-related morbidity and mortality may seem like a daunting task, there is a solution. Outcomes studies of MTMS provided by pharmacists working in interprofessional care teams have consistently demonstrated improved clinical outcomes, reduced healthcare expenditures, and favorable return on investment [6,7,8,9,10,11]. The fact that the U.S. healthcare system is leaving an antiquated fee-for-service reimbursement system behind in favor of value-based healthcare delivery and financing is good news for individuals who take medications. This project was designed to address community-based care system needs by ensuring service level expectations of MTM services provided by community pharmacists, equipping pharmacists to function effectively in SPCC community-based healthcare teams, and integrating the electronic documentation of community pharmacists providing MTMS into the SPCC HIT infrastructure. 

Future research and pharmacy/pharmacist opportunities will be created by understanding the MTMS service level expectations of community-based care teams, establishing tools and resources for community pharmacists to function effectively in high-performing health teams, and by integrating the electronic documentation of community pharmacists providing MTMS into HIT infrastructure. 

The health care industry is implementing a range of approaches for succeeding in risk-bearing and outcomes-based financing to shift payments from volume to value [23]. Competencies for effective and efficient team-based medication management in which pharmacists are integrated into community-based health teams is essential to achieving this value-based care model objective. The experiences and results of this study are expected to make an important contribution to building community-based medication management toolkits of the future.

## 5. Conclusions

Pharmacist integration in healthcare teams in hospitals, skilled nursing facilities, and clinics has grown steadily over the past 30 years. Community pharmacist integration in healthcare teams has unique challenges owing largely to distance, information access, and reimbursement. As healthcare delivery and financing transitions from expensive volume-based services and procedures in hospitals, emergency departments and large facilities, to community-based care delivery and value-based financing, there is a new opportunity for community pharmacists to develop patient care practices. This feasibility study of community pharmacist integration was enabled by a community-based ACO with strong support for community pharmacist integration in new care delivery and information exchange models, as well as the presence of a pharmacist reimbursement system for MTM services.

Although the daunting task of bi-directional exchange of health information between health systems and community pharmacists persist, there were small steps forward in terms of using Direct Secure Messaging to communicate with other community health team members, as well as the emergence of new Pharmacy eCare Plan standards for integrating pharmacists’ MTM documentation into electronic health records. A significant lesson learned in this feasibility study is the importance of understanding the skills and abilities of all other care providers in a community and then working together in coordinated action similar to high performing health teams in large medical centers. Equally important are lessons learned from unsuccessful sites in that removing barriers of reimbursement, billing, collaborative prescriber relationships, and technology access are insufficient to advance community pharmacy practice without also guiding management support for social and cultural transformation so that the business of patient care can coexist and complement the prescription dispensing business.

## Presentation

The preliminary results of this feasibility study were presented at the Minnesota Rural Health Conference in Duluth, MN on 20 June 2016.
